# Enhancement of Oral Tolerance Induction in DO11.10 Mice by *Lactobacillus gasseri* OLL2809 via Increase of Effector Regulatory T Cells

**DOI:** 10.1371/journal.pone.0158643

**Published:** 2016-07-29

**Authors:** Ayako Aoki-Yoshida, Kiyoshi Yamada, Satoshi Hachimura, Toshihiro Sashihara, Shuji Ikegami, Makoto Shimizu, Mamoru Totsuka

**Affiliations:** 1 Department of Applied Biological Chemistry, The University of Tokyo, Yayoi, Bunkyo-ku, Tokyo, Japan; 2 National Institute of Livestock and Grassland Science, National Agriculture and Food Research Organization, Tsukuba, Ibaraki, Japan; 3 Research Center for Food Safety, Graduate School of Agricultural and Life Sciences, The University of Tokyo, Tokyo, Japan; 4 Division of Research and Development, Meiji Co. Ltd., Odawara, Kanagawa, Japan; Wayne State University, UNITED STATES

## Abstract

Food allergy is a serious problem for infants and young children. Induction of antigen-specific oral tolerance is one therapeutic strategy. Enhancement of oral tolerance induction by diet is a promising strategy to prevent food allergy in infants. Thus, in this study, we evaluate the effect of probiotic *Lactobacillus gasseri* OLL2809 (LG2809) on oral tolerance induction in a mouse model. The degree of oral tolerance induction was evaluated by measuring the proliferation and level of IL-2 production of splenic CD4^+^ T cells from DO11.10 mice fed ovalbumin (OVA) alone or OVA with LG2809. Oral administration of LG2809 significantly decreased the rate of proliferation and IL-2 production by CD4^+^ T cells from OVA-fed mice. LG2809 increased a ratio of CD4^+^ T-cell population, producing high levels of IL-10 and having strong suppressive activity. Moreover, LG2809 increased a ratio of plasmacytoid dendritic cells (pDCs) among the lamina propria (LP) in small intestine. When used as antigen presenting cells to naïve CD4^+^ T cells from DO11.10 mice, LP cells from BALB/c mice fed LG2809 induced higher IL-10 production and stronger suppressive activity than those from non-treated mice. These results suggest that oral administration of LG2809 increases the population of pDCs in the LP, resulting in the enhancement of oral tolerance induction by increasing the ratio of effector regulatory T cells. LG2809 could, therefore, act as a potent immunomodulator to prevent food allergies by promoting oral tolerance.

## Introduction

Probiotics were defined as “live microorganisms which, when administered in adequate amounts, confer a health benefit to the host” by Food and Agricultural Organization of the United Nations /World Health Organization [1]. A growing body of evidence is accumulating to show that administration of probiotics modulate intestinal immunity, improve the balance of the gut microbiota, enhance the recovery of a disturbed gut mucosal barrier, and prevent microbial translocation [2, 3]. *Lactobacillus gasseri* OLL2809 (LG2809) is a probiotics that can reduce serum antigen-specific IgE levels in mice, and reduce the symptoms of Japanese cedar pollinosis [4–7]. We have previously shown *in vitro* that LG2809 suppresses proliferation of CD4^+^ T cells through a myeloid differentiation primary response gene 88 (MyD88)-dependent signaling pathway and that its RNA suppresses the delayed-type hypersensitivity response *in vivo* [8]. Hence, LG2809 is likely to have the potential to modulate various immune responses.

In recent years, food allergy has become a serious problem in infants and young children. The general treatment is to remove food allergens from the diet [9]. However, because milk and egg, the most frequent allergens in most countries, are nutritionally important sources of dietary proteins, especially for infants, removal of allergenic foods leads to an increased risk of undernutrition [10]. In addition, the developmental progression of allergic disease during early childhood is often known as the atopic march [11]. Therefore, it is beneficial for infants to achieve an early remission from food allergy.

Oral tolerance is the antigen-specific immune hyporesponsiveness to protein antigens repeatedly administered by the oral route [12]. Induction of antigen-specific oral tolerance is a promising strategy for treating food allergy [13]. Thus, it would be useful to enhance oral tolerance induction for an early remission from or to prevent food allergy in infants. Oral tolerance is mediated by multiple mechanisms, such as anergy, clonal deletion, and regulatory T-cell induction [14]. Antigen-specific T-cell anergy by oral tolerance induction was demonstrated by the transfer of T cells and B cells from orally tolerized mice into SCID mice [15]. The clonal deletion process occurs by apoptosis of antigen-specific CD4^+^ T cells [16], which in oral tolerance induction is mediated by signaling via Fas antigen and p55 tumor necrosis factor (TNF) receptor [17, 18]. Various regulatory T cells are induced by oral tolerance induction. Oral administration of myelin basic protein induces regulatory transforming growth factor (TGF)-β-secreting T cells in Peyer's patches of mice [19]. Oral tolerance induction in ovalbumin (OVA)-specific T-cell receptor (TCR) transgenic mice (DO11.10 mice) leads to an increase in regulatory T cells, and they produce high levels of IL-10 and exert suppressive activity [20]. There are several reports of dendritic cell (DC) involvement in the induction of oral tolerance and T-cell differentiation [21–24]. DCs capture dietary antigens in the intestinal mucosa and present them to T cells. DCs are a heterogeneous population of leucocytes that act as professional antigen-presenting cells (APCs) [25]. In particular, DCs in the intestinal lamina propria (LP) have been shown to play an essential role in oral tolerance induction [22, 26, 27]. There are two classes of DCs, myeloid (mDC) and plasmacytoid (pDC), which are functionally different; they differ in cytokine/chemokine secretion, expression of cell surface markers, and T-cell-polarizing ability [18, 26, 28–32]. Interestingly, recent studies have shown that nutrients and food antigens can alter DC phenotypes and behaviors [33–35], suggesting that intestinal luminal contents are directly involved in modulating mucosal DC function.

The intestinal microbiota play important roles in oral tolerance induction and its long-term persistence [36, 37]. Several studies suggest that specific-pathogen free (SPF), but not germ-free mice, are susceptible to induction of oral tolerance [38–40]. Colonization of gut bacteria can restore oral tolerance in germ-free mice, and the effect of probiotics is dependent on the bacterial strain [38, 39]. *Lactobacillus casei* potentiates the induction of oral tolerance and suppresses the T helper (Th)1-type immune responses of inflammation in a SPF-rat model of experimental arthritis [41]. Furthermore, probiotic treatment can alter DC phenotype and function. Feeding with the probiotic strain *Lactobacillus paracasei* subsp. paracasei NTU 101 [42] or with *L*. *casei* DN-114001-fermented milk [42, 43] results in the up-regulation of the antigen-presenting ability of DCs. In addition, administration of the probiotic nutrient supplement VSL#3 has been shown to increase the proportion of mDCs within the LP [27]. These findings suggest that probiotics would affect oral tolerance induction via alteration of the LP DC phenotype and its function. However, the effect of probiotics on oral tolerance remains largely unknown.

In the present study, we investigated the effect of oral administration of LG2809 on the induction of oral tolerance to OVA by examining the proliferative response, IL-2 production, and suppressive activity of splenic CD4^+^ T cells from DO11.10 mice. To elucidate the mechanism of the enhancement of oral tolerance induction by LG2809, the effect of LG2809 on DC responses in the LP was examined.

## Materials and Methods

### Mice

Female BALB/c mice (8–10 weeks old) were obtained from CLEA Japan (Tokyo, Japan). Female DO11.10 T-cell receptor transgenic mice (8–10 weeks old) were transgenic for OVA323–339-specific and I-A^d^-restricted T-cell receptor αβ, with a BALB/c genetic background [44]. These mice were fed chow CE-2 (CLEA) ad libitum and housed in cages (6 mice per cage) maintained under a continuous 12 hour light: 12 hour dark cycle in conventional conditions at the Department of Applied Biological Chemistry, the University of Tokyo. Hair gloss of mice was checked daily to monitor their health. All of the mice used in this study were euthanized by cervical dislocation. All animal care and use protocols were conducted in accordance with the animal experimentation guidelines of the University of Tokyo. The research protocol was approved by the Animal Experimentation Committee of the University of Tokyo (Tokyo, Japan; permit number: P07-065, P08-242).

### Reagents

The following antibodies were used: fluorescein isothiocyanate (FITC)-conjugated anti-mouse CD4 (clone H129.19), anti-DO11.10 clonotypic TCR (KJ-1.26), anti-mouse CD44 (clone IM7), peridinin chlorophyll protein (PerCP)-conjugated anti-mouse CD4 (clone RM4-5), allophycocyanin (APC)-conjugated anti-mouse CD4 (clone 145-2C11), biotinylated anti-mouse CD3ε (clone 145-2C11), biotinylated anti-mouse CD62L (clone MEL14), biotinylated anti-mouse CD103 (clone M290), biotinylated anti-mouse CD11b (clone M1/70), and streptavidin-phycoerythrin (PE)-cyanine (Cy)5 and PE-anti-mouse Foxp3 (clone FJK-16s) were purchased from BD Biosciences (San Diego, CA, USA). FITC-conjugated anti-mouse CD11c (clone N418), PE-anti mouse CD45R/B220 (clone RA3-6B2), and biotinylated anti-mouse Ly-6G (clone RB6-8C5) were purchased from eBioscience (San Diego, CA, USA).

### Bacterial strain and preparation

LG2809 was provided by the Division of Research and Development, Meiji Co., Ltd. (Odawara, Japan). LG2809 was cultured in Lactobacilli MRS broth (DIFCO, Detroit, MI, USA) at 37°C for 18 hrs. Bacteria were harvested by centrifugation at 1,800 x *g* at 4°C for 15 mins, and then washed twice with 0.85% NaCl (saline). The cells were collected and suspended in saline at 5 mg dry weight/mL. The bacterial suspension was stored at -80°C until required.

### Oral tolerance induction and treatment of mice with LG2809

DO11.10 mice were administered saline or live LG2809 (1 mg/day) by gavage for 12 days. For the last 5 days, the mice were fed a solution of 20% OVA (Wako; Albumin, from Eggs, 012–09885) *ad libitum* instead of sterile water. To examine the effect of LG2809 on the phenotype and function of LPDCs, BALB/c mice were orally administered LG2809 (1 mg/day) by gavage for 7 days.

### Cell preparation and culture conditions

Cells were cultured in RPMI1640 (Nissui Pharmaceutical, Tokyo, Japan) containing 10% fetal calf serum (FCS; GIBCO, Grand Island, NY, USA), 2 g/L NaHCO_3_, 100 U/mL penicillin, 100 μg/mL streptomycin, 50 μM 2-mercaptoethanol, and 300 mg/L L-glutamine at 37°C in 5% CO_2_ in air.

Mouse spleen (SPL) CD4^+^ T cells were isolated as previously described [45] using magnetic activated-cell sorting (MACS) positive selection with CD4 microbeads (Miltenyi Biotec, Bergish Gladbach, Germany) according to the manufacturer’s instructions. CD4^+^ CD25^-^ T cells were purified using a CD4^+^ CD25^+^ regulatory T-cell isolation kit (Miltenyi Biotec).

T-cell-depleted splenocytes were isolated by MACS negative selection using Thy-1.2 microbeads (Miltenyi Biotec), and the isolated cells were used as APCs.

Preparation of FACS-sorted cells from DO11.10 mice was performed as follows: MACS- sorted CD4^+^ T cells were washed with PBS. Then the cells (2–4 x 10^7^ cells/mL) were incubated with biotinylated anti-CD62L antibody in PBS/1%FCS buffer for 20 mins on ice. After the cells were washed, they were incubated with anti-KJ-1.26-FITC, PE-conjugated anti-CD44 antibody, and streptavidin-PE-Cy5 for 20 mins on ice. KJ-1.26^+^ CD62L^high^ CD44^high^ and KJ-1.26^+^ CD62L^low^ CD44^high^ T cells were isolated from CD4^+^ T cells of LG2809 and OVA-fed mice using a FACS Vantage SE (BD Biosciences). The purity of the sorted populations was routinely >95%.

LP cells from BALB/c mice were isolated from the small intestines as follows: Peyer’s patches were removed from the small intestines to prevent their lymphocyte contamination in LP cells. After cutting open the intestines longitudinally, they were cut into pieces of approximately 4 cm and washed with Ca- and Mg-free Hanks’ balanced salt solution (HBSS; GIBCO) containing 0.04% NaHCO_3_, 5 mM EDTA (pH 8.0) and 5% FCS with shaking at 37°C in 5% CO_2_ in air for 20 mins, three times. The supernatant was discarded following filtration with gauze, and the intestines were minced into 5 mm pieces and treated with 1 mg/mL collagenase IV (Sigma-Aldrich, St Louis, MO, USA) in HBSS containing Ca, Mg, and 5% FCS (HBSS (+)) in a 100 mL flask with gentle stirring at 37°C in 5% CO_2_ in air for 60 mins. After collagenase treatment, the preparation was filtered with gauze and the cells were washed with HBSS (+) followed by centrifugation at 25°C, 300 x *g* for 5 mins. The supernatant was then removed and 3 mL of 100% Percoll (Amersham Biosciences, Uppsala, Sweden) was added to the cell pellets and made up to 10 mL with HBSS (+) (a 30% Percoll concentration). The cell suspension was mixed gently and centrifuged at 25°C, 580 x *g* for 20 mins. All but 1 mL of the supernatant was then removed, the cells were resuspended, and 4.1 mL of 100% Percoll and 10 mL of RPMI containing 10% FCS was added (a 44% Percoll concentration). Next, 2 mL of 70% Percoll was carefully added beneath the cell suspension and centrifuged at 20°C, 580 x *g* for 20 mins. Finally, the cells located at the interface between the 44% and 70% Percoll fractions were collected as LP cells.

CD3^-^ cells among the LP cells were isolated using a MACS LD column (Miltenyi Biotec). The CD3-negative fraction was used as an APC-enriched fraction that included DCs. SPL CD4^+^ CD25^-^ T cells from DO11.10 mice were stimulated with OVA323–339 and CD3^-^ LP cells, and the activated CD4^+^ T cells were purified using MACS positive selection with CD4 microbeads.

### CD4^+^ T-cell proliferation assay

After oral administration of LG2809, SPL cells were obtained from DO11.10 mice in each group. Isolated CD4^+^ T cells (1 x 10^5^ cells/well) and APCs (1 x 10^5^ cells/well) were cultured with OVA323–339 (1 μM) in 96-well flat-bottomed plates at 37°C in 5% CO_2_ in air. Proliferation of the CD4^+^ T cells was evaluated by measuring incorporation of ^3^H-thymidine (37 kBq/ well) (ICN Pharmaceuticals, Costa Mesa, CA, USA) added into the culture during the final 24 hrs of incubation.

### Cytokine analysis

Isolated CD4^+^ T cells (1 x 10^5^ cells/well) and APCs (1 × 10^5^ cells/well) were cultured with OVA323–339 (1 μM) in U-bottomed 96-well plates at 37°C in 5% CO_2_ in air. After incubation for 2 days, culture supernatants were collected.

Determination of the IL-2 level in the culture supernatants was performed by a sandwich enzyme-linked immunosorbent assay (ELISA), as previously described [46]. Briefly, anti-mouse IL-2 (clone JES6-1A12, BD PharMingen, San Diego, CA, USA) was coated on ELISA plates. After washing and blocking the plates, samples and standards were added. After washing, biotinylated anti-mouse IL-2 (clone JES6-5H4, BD PharMingen) was added. The wells were washed, and streptavidin-conjugated alkaline phosphatase (Zymed, South San Francisco, CA, USA) was added. The wells were washed and incubated with disodium 4-nitrophenylphosphate hexahydrate solution. Optical densities were read at 405 nm on a BIO-RAD Model 550 Microplate Reader (Bio-Rad, Hercules, CA, USA). The IL-10 level in the culture supernatants was measured by sandwich ELISA using Mouse Opt EIA kits (BD Biosciences), as described previously [47].

### Flow cytometric analysis

Flow cytometric analysis was performed using a FACS LSR with CellQuest software (BD Biosciences). The cells were harvested and washed with PBS containing 1% fetal calf serum and 0.1% NaN_3_ (FACS buffer). The cells were incubated with anti-CD16/CD32 monoclonal antibody (mAb; clone 2.4G2, BD PharMingen) on ice to block non-specific binding to Fc receptors. Then, the cells were stained with the appropriate PerCP-, FITC-, or PE-conjugated antibodies or biotinylated antibody for 30 mins on ice. When stained with the biotinylated antibody, the cells were further stained with streptavidin-APC conjugated antibody for 30 mins on ice. The cells were analyzed by flow cytometry.

For apoptotic analysis, the cells were washed in FACS buffer and incubated with anti-CD16/CD32 mAb (BD PharMingen) on ice. The cells were then stained with biotinylated CD4 antibodies for 30 mins on ice, followed by staining with anti-KJ-1.26-FITC (BD PharMingen) and anti-CD4-APC (BD PharMingen) for 30 mins on ice. Cells were further stained using an Annexin V-PE Apoptosis Detection kit I (BD Biosciences) and analyzed.

For intracellular analysis of the expression of Foxp3, the cells were fixed with Fixation/Permeabilization Concentrate (eBioscience) and Fixation/Permeabilization Diluent (eBioscience) overnight at 4°C. The cells were washed with Permeabilization Buffer (eBioscience) and incubated with anti-CD16/CD32 mAb (BD PharMingen) on ice. Then the cells were stained with anti-Foxp3-PE mAb (clone FJK-16s; BD Biosciences) for 30 min on ice, and the cells were subjected to the flow cytometric analysis.

### Assays for T-cell suppressive activity

To determine the T-cell suppressive activity of the groups of splenic CD4^+^ T cells, i.e. CD62L^high^ CD44^high^ CD4^+^ T cells and CD62L^low^ CD44^high^ CD4^+^ T cells from DO11.10 mice fed OVA and LG2809, and the CD4^+^ T cells differentiated *in vitro* through activation with antigen-presentation by CD3^-^ LP cells, the ability of these groups of T cells to inhibit IL-2 production by responder SPL CD4^+^ CD25^-^ T cells was measured. CD4^+^ T cells (1 x 10^5^ cells, 5 x 10^4^ cells, or 2.5 x 10^4^ cells/well) were co-cultured with CD4^+^ CD25^-^ T cells from untreated DO11.10 mice (5 x 10^4^ cells/well), APCs (1 x 10^5^ cells/well), and 0.3 μM OVA323–339 in 96-well U-bottomed plates at 37°C in 5% CO_2_ in air. Then the amount of IL-2 in the culture supernatants was analyzed by ELISA.

### Statistical analysis

All experimental data were expressed as the mean ± standard deviation (SD). Statistical differences were analyzed by Student’s *t*-tests.

## Results

### Oral administration of live LG2809 enhanced oral tolerance induction in DO11.10 mice

**Fig 1:**
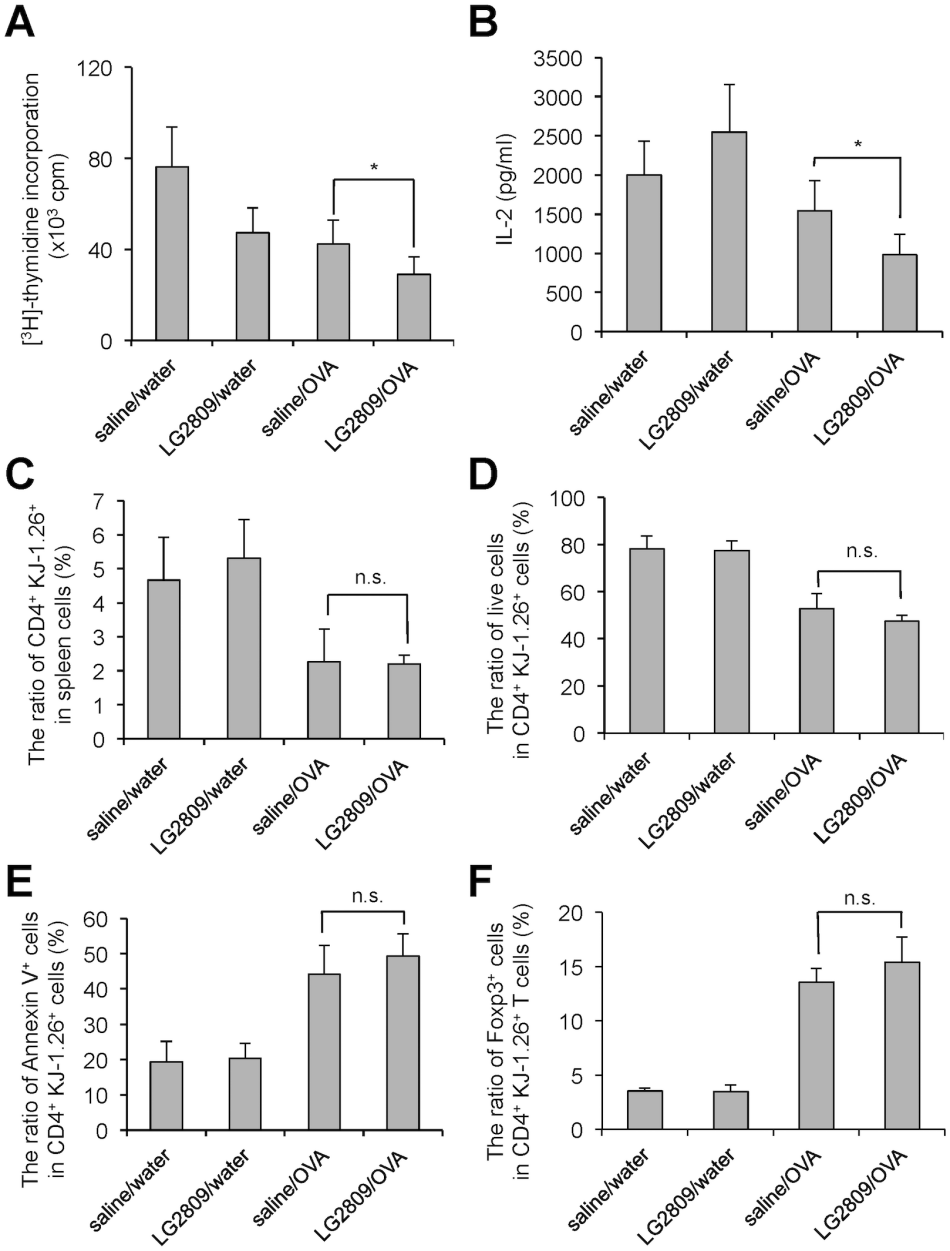
Oral administration of live LG2809 enhanced oral tolerance induction in DO11.10 mice via non-apoptotic pathways. DO11.10 mice were fed live LG2809 (1 mg/day; treated) or 0.85% NaCl (saline; untreated) for 12 days. For the last 5 days, the mice did (oral tolerance group) or did not (control group) have OVA added to the drinking water. According to feeding regimes, the groups were as follows: saline/water (untreated control group), saline/OVA (untreated oral tolerance group), LG2809/water (treated control group), and LG2809/OVA (treated oral tolerance group). SPL CD4^+^ T cells from each group were cultured with APCs and 1 μM OVA323–339. (A) The cultures were pulsed with [^3^H]-thymidine for the last 24 hrs of the 96 hrs culture period and [^3^H]-thymidine incorporation was measured. (B) IL-2 in the supernatant after 48 hrs in culture was measured by ELISA. (C, D and E) Whole SPLs from DO11.10 mice of each group were stained with anti-CD4-PerCP, anti- KJ-1.26-FITC, and annexin V-PE. The ratio of apoptotic cells was determined by flow cytometric analysis. The ratio of CD4^+^ KJ-1.26^+^ cells (C), of live (CD4^+^ KJ-1.26^+^ annexin V^-^ 7-AAD^-^) cells (D), of CD4^+^ KJ-1.26^+^ annexin V^+^ apoptotic cells (E) and CD4^+^ KJ-1.26^+^ Foxp3^+^ cells (F) is shown for each group. Data are shown as the means ± SD (n = 6). Data are representative of two independent experiments. Statistical differences were analyzed by Student’s *t*-test and were considered significant (*) when *p* was <0.05. n.s., not significant.

Female DO11.10 mice were administered 1 mg of live LG2809 in saline or saline alone by gavage every day for 12 days. The mice were fed 20% OVA in drinking water *ad libitum* for the last 5 days. Consistent with a phenotype of oral tolerance, the proliferation rate and level of IL-2 secretion were lower in SPL T cells from OVA-fed mice (saline/OVA group) than in T cells from untreated mice (saline/water group; Fig 1A and 1B). Oral administration of live LG2809 and OVA (LG2809/OVA group) further decreased proliferation and IL-2 secretion to levels significantly below those of the saline/OVA group, indicating that oral administration of LG2809 enhanced oral tolerance induction. Clonal deletion of antigen-specific CD4^+^ T cells is known to occur with oral tolerance induction [16]. Thus, we examined whether oral administration of LG2809 affected apoptosis of OVA-specific T cells. OVA-specific T cells from DO11.10 mice can be detected with the KJ-1.26 mAb specific for the transgenic TCR. The ratio of CD4^+^ KJ-1.26^+^ T cells among SPL cells and the ratio of viable cells (annexin V^-^ 7-AAD^-^) among CD4^+^ KJ-1.26^+^ T cells from DO11.10 mice fed OVA were lower compared to those found in DO11.10 mice fed no OVA, and the ratio of annexin V^+^ cells among CD4^+^ KJ-1.26^+^ cells from the saline/OVA group was higher compared to those from saline/water group. However, there was no significant difference between the groups fed or not fed LG2809 (Fig 1C–1E). These results suggest that clonal deletion of antigen-specific T cells is not responsible for the decrease in proliferation and IL-2 production by SPL CD4^+^ T cells induced by feeding LG2809. Since it has been reported that Tregs play a crucial role for the induction of oral tolerance [19], and thus we examined the effect of LG2809 on the induction of Foxp3^+^ Treg. Although the ratio of Foxp3^+^ cells among CD4^+^ KJ-1.26^+^ T cells was increased by oral tolerance induction, no effect was observed by the administration of LG2809 (Fig 1F).

### Oral administration of live LG2809 enhanced IL-10 secretion and T-cell suppressive activity in splenocytes from orally tolerized mice

**Fig 2:**
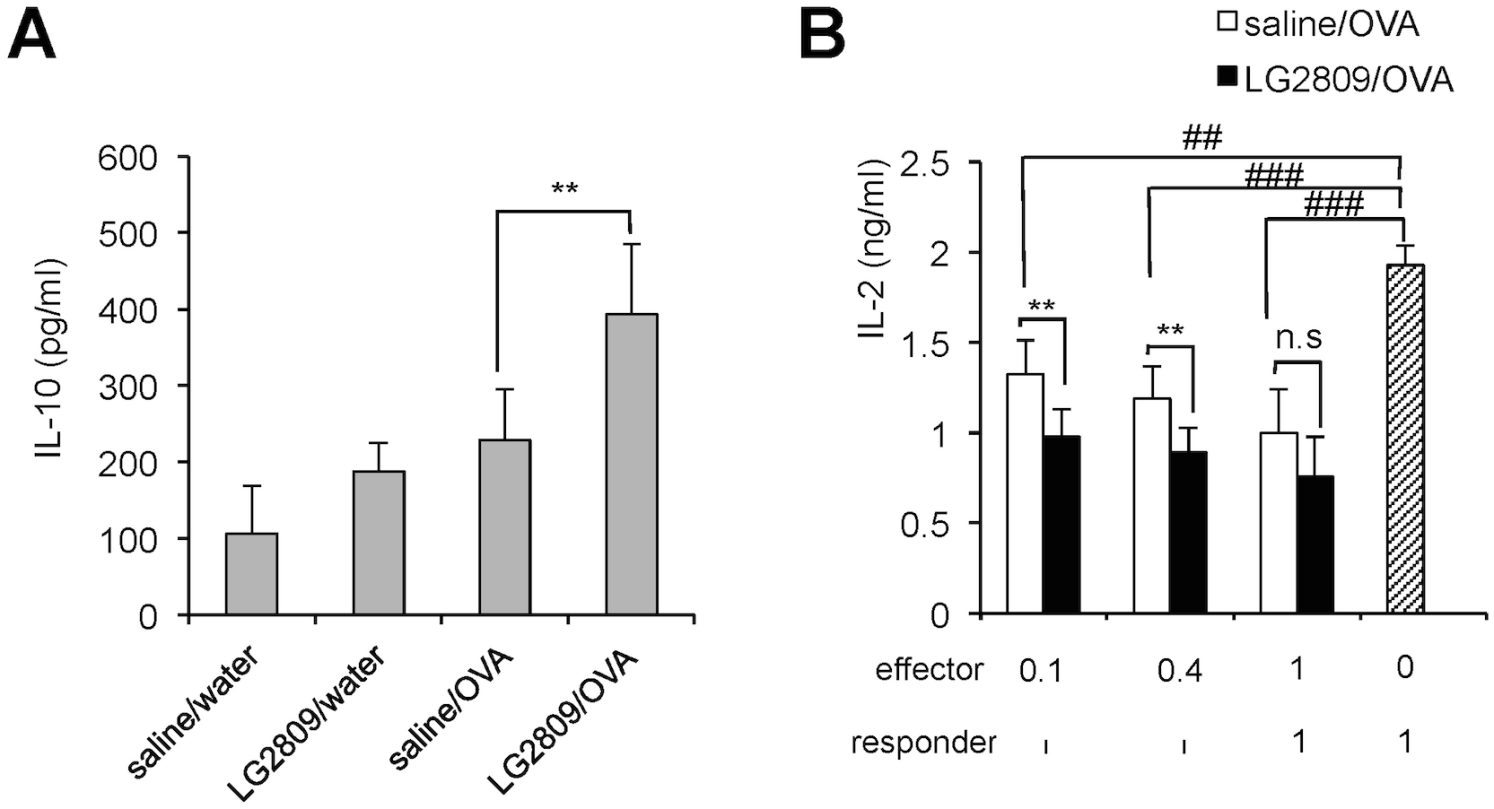
LG2809 enhanced IL-10 secretion and T-cell suppressive activity in splenocytes from orally tolerized mice. DO11.10 mice were treated as described in the legend to Fig 1. (A) SPL CD4^+^ T cells from DO11.10 mice of each group were cultured with APCs and 1 μM OVA323–339. IL-10 in the supernatant after 48 hrs in culture was measured by ELISA. (B) SPL CD4^+^ T cells from DO11.10 mice of the saline/OVA or LG2809/OVA groups (effector cells) were cultured with APCs, 1 μM OVA323–339, and responder DO11.10 CD4^+^ CD25^-^ T cells at the ratio indicated in the figure. IL-2 in the supernatant was measured by ELISA. Data are shown as means ± SD. (n = 6). Data are representative of two independent experiments. Statistical differences were analyzed by Student’s *t*-test. *p<0.05 and **p<0.01, significantly different vs. saline/OVA group; ^##^*p*<0.01 and ^###^*p*<0.001, significantly different vs. responder DO11.10 CD4^+^ CD25^-^ T cells alone.

Since IL-10-secreting T cells are induced in the SPL of orally tolerized mice [20, 48], IL-10 secretion in the culture medium of the SPL CD4^+^ T cells from the different groups was examined. Consistent with previous reports, the level of IL-10 secretion by orally tolerized T cells was higher than that of the control cells. Furthermore, oral administration of LG2809 significantly enhanced IL-10 secretion from the T cells of OVA-fed mice (Fig 2A). Next, we examined the effect of LG2809 on suppressive activity of CD4^+^ T cells from OVA-fed DO11.10 mice. SPL CD4^+^ T cells from each test group of mice, responder CD4^+^ CD25^-^ T cells from untreated DO11.10 mice, and APCs were cultured in the presence of OVA323–339, and then the levels of IL-2 secretion were examined. As shown in Fig 2B, SPL CD4^+^ T cells from the saline/OVA group caused a decrease in the level of IL-2 secretion from the responder CD4^+^ CD25^-^ T cells. Moreover, feeding LG2809 significantly enhanced the suppressive effect of the T cells on IL-2 secretion.

### Oral administration of live LG2809 increased the ratio of CD62L^low^ CD44^high^ CD4^+^ T cells with IL-10-producing and suppressive activities

**Fig 3:**
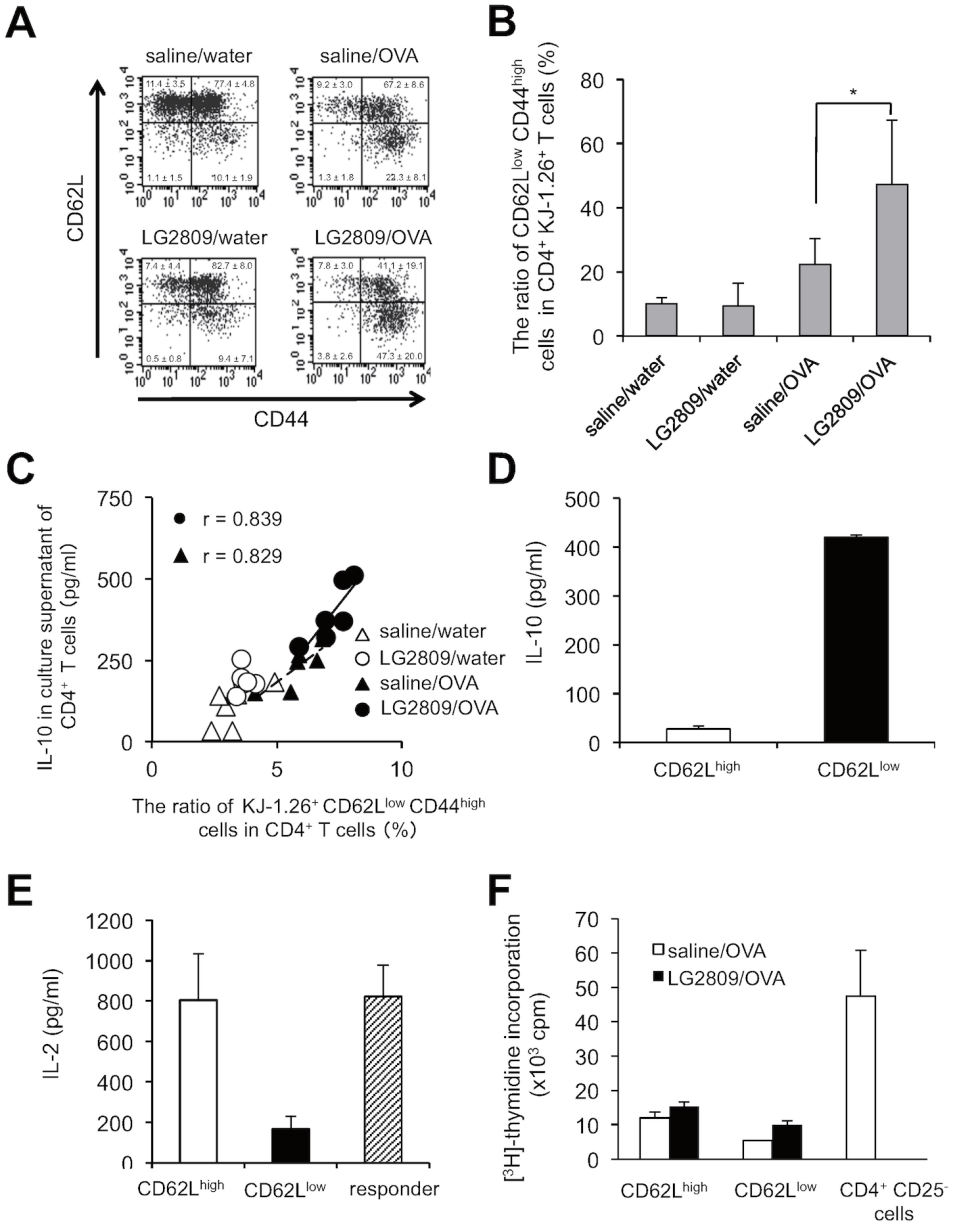
LG2809 increased the ratio of CD62L^low^ CD44^high^ CD4^+^ T cells with IL-10-producing and suppressive activities. DO11.10 mice were treated as described in the legend to Fig 1. (A and B) Whole SPLs from DO11.10 mice of each group were stained with anti-CD4-PerCP, KJ-1.26-FITC, anti-CD44-PE, and anti-CD62L-APC. The expression of CD44 and CD62L on CD4^+^ KJ-1.26^+^ T cells was analyzed by flow cytometric analysis. The mean ratio of CD62L^low^ CD44^high^ cells in CD4^+^ KJ-1.26^+^ T cells is shown (B). Data are shown as means ± SD (n = 6). Data are representative of two independent experiments. Statistical differences were analyzed by Student’s *t*-test. *p<0.05. (C) The correlation between the concentration of IL-10 in the culture supernatant of CD4^+^ T cells shown in Fig 1C and the ratio of KJ-1.26^+^ CD62L^low^ CD44^high^ cells among CD4^+^ T cells. Closed circles, LG2809/OVA group; open circles, LG2809/water group; closed triangles, saline/OVA group; open triangles, saline/water group. The continuous line is a linear regression for LG2809/OVA group and the dashed line is a linear regression for saline/OVA group. R, Pearson correlation coefficient. Data are representative of two independent experiments. (D) CD62L^high^ CD44^high^ and CD62L^low^ CD44^high^ cells sorted from CD4^+^ T cells isolated from DO11.10 mice of the LG2809/OVA group were cultured with APCs and 0.3 μM OVA323–339. After 48 hrs, IL-10 in the supernatant was measured by ELISA. (E) The sorted CD62L^high^ CD44^high^ and CD62L^low^ CD44^high^ CD4^+^ T cells from DO11.10 mice of the LG2809/OVA group were incubated with responder CD4^+^ T cells from DO11.10 SPL (at a ratio of 1:1), plus APCs and 0.3 μM OVA323–339. IL-2 in the supernatant was measured by ELISA. (F) The sorted CD62L^high^ CD44^high^ and CD62L^low^ CD44^high^ CD4^+^ T cells from DO11.10 mice of the saline/OVA, LG2809/OVA group, and CD4^+^ CD25^-^ T cells were incubated with APCs and 0.3 μM OVA323-339. The cultures were pulsed with [^3^H]-thymidine for the last 24 hrs of the 96 hrs culture periods and [^3^H]-thymidine incorporation was measured. Data are representative of two independent experiments.

T cells can be divided into three phenotypes (naïve T cells, memory T cells, and effector T cells) according to the expression of CD44 and CD62L molecules, and therefore to further analyze the subsets involved in tolerance induction, we examined the expression of CD44 and CD62L in CD4^+^ KJ-1.26^+^ T cells from LG2809 and OVA-fed mice by flow cytometry. The results indicated that the ratio of CD62L^low^ CD44^high^ CD4^+^ T cells, considered to be the effector phenotype, was higher among SPL T cells from OVA-fed mice, compared to those from untreated mice. The ratio of this subset was significantly increased in the LG2809/OVA group compared to that in the saline/OVA group (Fig 3A and 3B). To elucidate the relationship between the effector T cells and IL-10 production, the ratio of KJ-1.26^+^ CD62L^low^ CD44^high^ cells among CD4^+^ T cells was plotted against IL-10 concentration in the culture supernatant of CD4^+^ T cells. We found a positive correlation between the ratio of KJ-1.26^+^ CD62L^low^ CD44^high^ cells among CD4^+^ T cells and the IL-10 concentration in the culture supernatant of CD4^+^ T cells in both saline/OVA and LG2809/OVA groups (Fig 3C). To assess whether CD62L^low^ CD44^high^ CD4^+^ T cells produce IL-10 and have suppressive activity, IL-10 production and suppressive activity of sorted CD62L^low^ CD44^high^ CD4^+^ T cells and CD62L^high^ CD44^high^ CD4^+^ T cells isolated from DO11.10 mice of LG2809/OVA group were also investigated. Cultured sorted CD62L^low^ CD44^high^ CD4^+^ T cells secreted high concentrations of IL-10 into the supernatant (Fig 3D). Furthermore, sorted CD62L^low^ CD44^high^ T cells had stronger suppressive activity against co-cultured responder T cells than CD62L^high^ CD44^high^ CD4^+^ T cells (Fig 3E). Proliferative response of the sorted cells was also examined. CD62L^low^ CD44^high^ CD4^+^ T cells and CD62L^high^ CD44^high^ CD4^+^ T cells proliferated much more weakly than CD4^+^ CD25^-^ T cells, indicating that they were both anergic cells (Fig 3F). These results suggest that the enhanced IL-10 production and suppressive activity of SPL CD4^+^ T cells from LG2809-fed orally tolerized mice is caused by an increase in the ratio of CD62L^low^ CD44^high^ CD4^+^ T cells.

Next, we plotted the ratio of KJ-1.26^+^ CD62L^low^ Foxp3^+^ cells among CD4^+^ T cells or that of KJ-1.26^+^ CD62L^low^ Foxp3^+^ cells against IL-10 concentration in the culture supernatant of each population of CD4^+^ T cells, respectively (S1 Fig). Interestingly, we found very good positive correlation between the ratio of KJ-1.26^+^ CD62L^low^ Foxp3^-^ cells among CD4^+^ T cells and the IL-10 concentration in the culture supernatant of CD4^+^ T cells in LG2809/OVA group. It is known that not only Foxp3 Tregs but also Foxp3 negative T regulatory type 1 (Tr1) cells are induced in oral tolerance [49]. Our results suggested that LG2809 might increase Tr1 cells but not Foxp3 Tregs in orally tolerized mice.

### Oral administration of live LG2809 increased the population of pDCs in the LP

**Fig 4:**
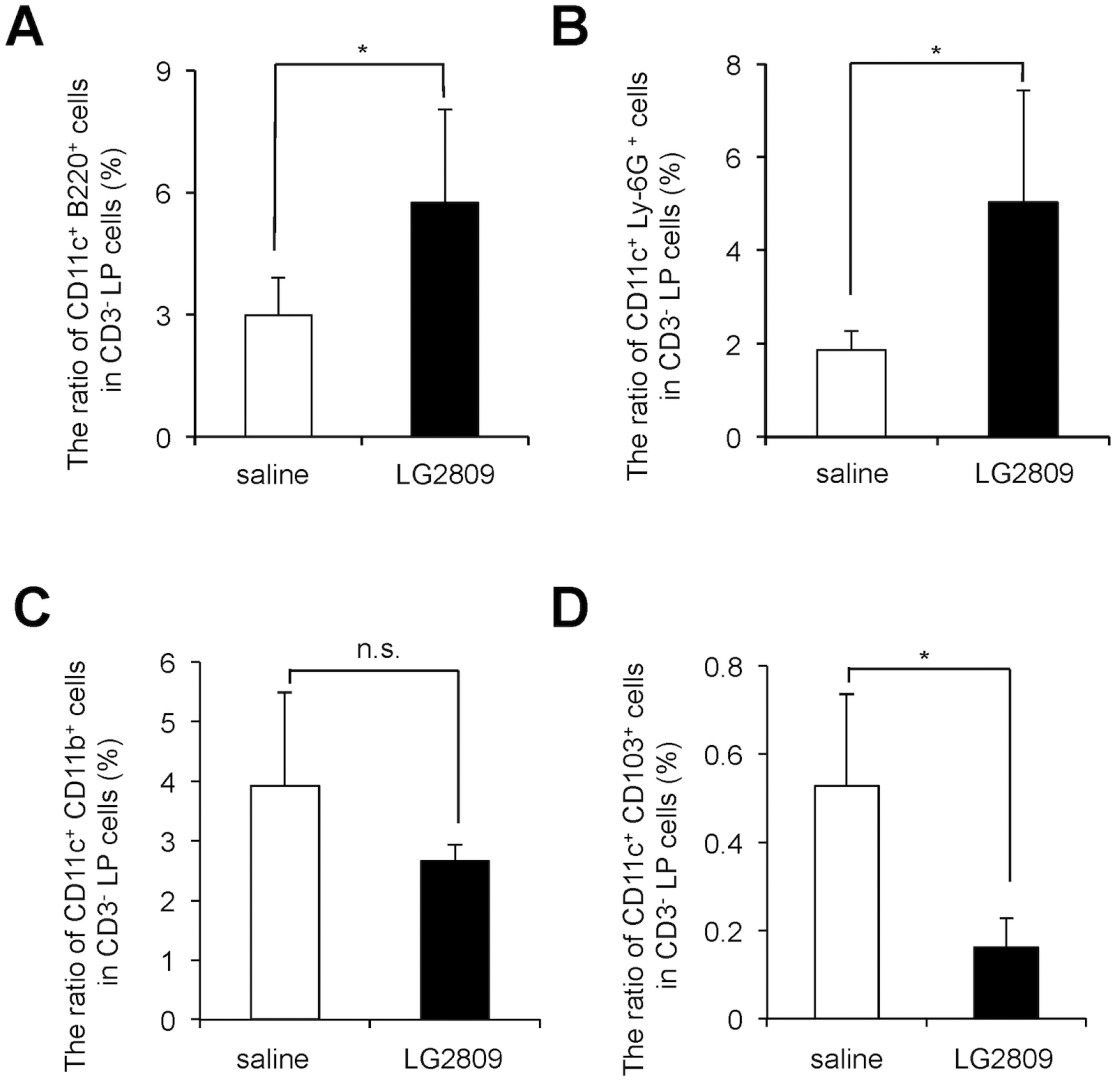
LG2809 enhanced plasmacytoid dendritic cells in the lamina propria. BALB/c mice were fed live LG2809 (1 mg/day) or saline for 7 days. The ratio of CD11c^+^ B220^+^ (A), CD11c^+^ Ly-6G^+^ (B), CD11c^+^ CD11b^+^ (C), and CD11c^+^ CD103^+^ (D) cells among CD3^-^ LP cells was determined by flow cytometric analysis. Data are shown as the means ± SD. (n = 4). Data are representative of two independent experiments. Statistical differences were analyzed by Student’s *t*-test. *p<0.05. n.s., not significant.

The two classes of functionally different DCs, mDC and pDC, are defined by the surface expression of CD11c, CD11b and CD103, and CD11c, B220 and Ly-6G, respectively [27, 30, 31, 50, 51]. DCs in the LP play important roles in oral tolerance induction [22, 26, 27], and nutrients and food antigens can alter the DC phenotype [33–35]. To investigate whether oral administration of LG2809 affects DCs in the LP, we examined the expression of DC surface molecules by LP cells of BALB/c mice fed live LG2809. BALB/c mice were fed LG2809 or saline for 7 days, and CD3^-^ LP cells were then isolated and analyzed for the expression of CD11c, B220, Ly-6G, CD11b and CD103 by flow cytometry (Fig 4). Feeding LG2809 significantly increased the ratio of CD11c^+^ B220^+^ and CD11c^+^ Ly-6G^+^ cells among the CD3^-^ LP cells (Fig 4A and 4B). On the other hand, LG2809 had no effect on the ratio of CD11c^+^ CD11b^+^ cells (Fig 4C) but significantly reduced the ratio of CD11c^+^ CD103^+^ cells among the CD3^-^ LP cells (Fig 4D). pDCs express CD11c, B220 and Ly-6G, and mDCs express CD11c, CD11b and CD103. Thus, the oral administration of LG2809 had led to an increase in the ratio of pDCs in the LP.

### Regulatory CD4^+^ T cells differentiated through activation with cells from the LP of mice fed LG2809 had stronger T-cell suppressive activity

**Fig 5:**
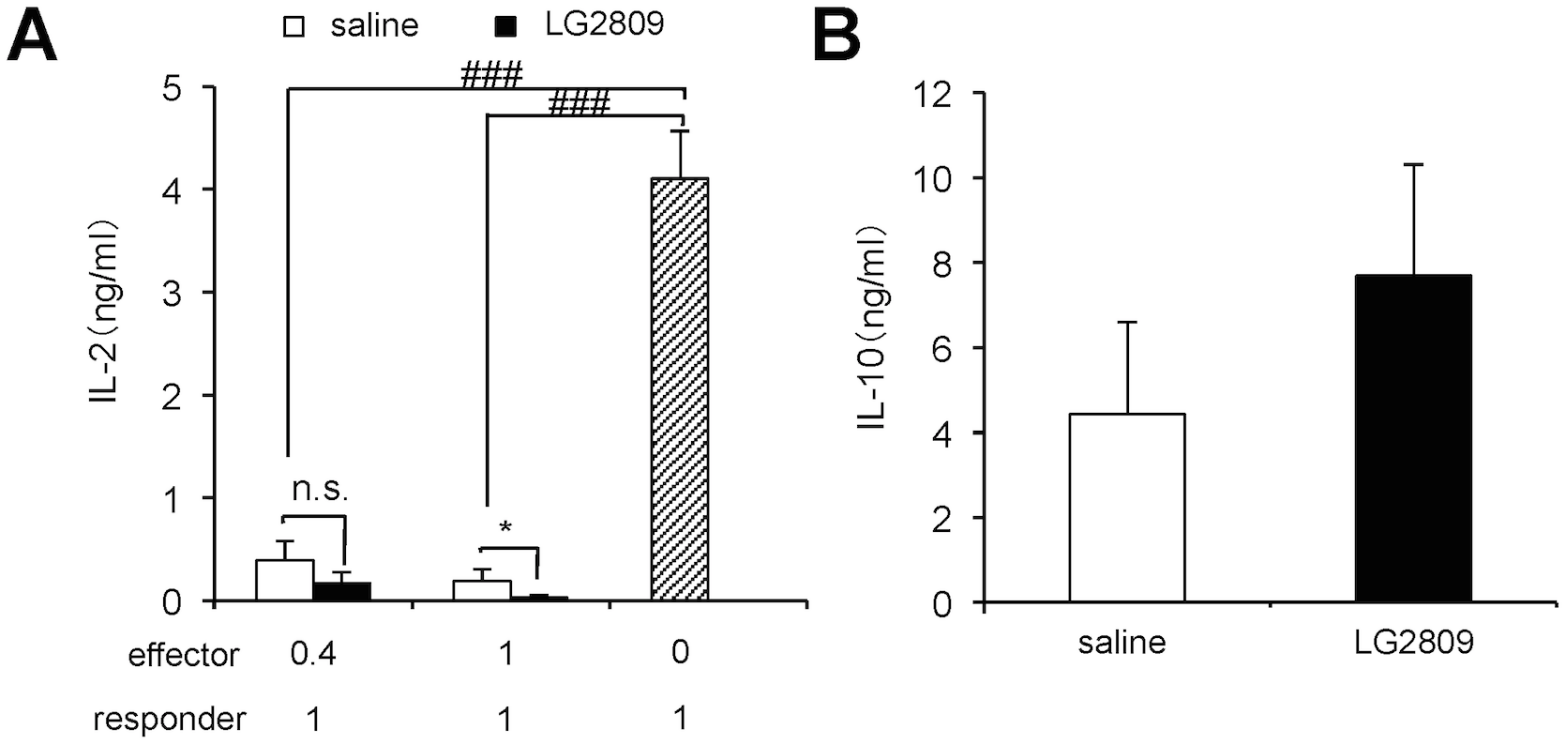
CD4^+^ T cells differentiated with lamina propria APCs from mice fed LG2809 had stronger suppressive activity. (A and B) BALB/c mice were fed live LG2809 (1 mg/day) or saline for 7 days. CD4^+^ CD25^-^ T cells from DO11.10 mice were cultured with 1 μM OVA323–339 and CD3^-^ LP cells from BALB/c mice fed LG2809 or saline as APCs. After 72 hrs, the CD4^+^ T cells were purified by MACS. The activated CD4^+^ T cells were incubated with responder CD4^+^ CD25^-^ T cells from DO11.10 SPL cells (at ratios of 0.4:1 and 1:1), plus APCs and 1 μM OVA323–339. After 48 hrs, IL-2 in the supernatant was measured by ELISA (A). The activated CD4^+^ T cells were incubated with APCs and 1 μM OVA323–339. After 48 hrs, IL-10 in the supernatant was measured by ELISA (B). Data are shown as means ± SD (n = 4). Data are representative of two independent experiments. Statistical differences were analyzed by Student’s *t*-test. *p<0.05, significantly different vs. saline group; n.s., not significant.; ^###^*p*<0.001, significantly different vs. responder DO11.10 CD4^+^ CD25^-^ T cells alone.

We next investigated whether LG2809 administration altered the antigen-presenting function of the LP cells. CD3^-^ LP cells were prepared from BALB/c mice administered saline or LG2809 for 7 days. SPL CD4^+^ CD25^-^ T cells from DO11.10 were stimulated with OVA323–339 and CD3^-^ LP cells as APCs. The CD4^+^ T cells activated with the antigen and CD3^-^ LP cells from mice fed LG2809 had significantly stronger suppressive activity (Fig 5A). In addition, the differentiated CD4^+^ T cells through activation with CD3^-^ LP cells from LG2809-fed mice produced marginally higher levels of IL-10 (Fig 5B), but no difference was found in the ratio of Foxp3^+^ cells (data not shown). These results suggest that LG2809 had altered the antigen-presenting function of LP cells and enhanced the suppressive activity of differentiated T cells.

## Discussion

Intestinal microbiota have been proved to play critical roles in the induction of oral tolerance [52, 53]. However, little is known about the effect of orally administered probiotic bacteria on the induction of oral tolerance. In this study, we investigated the effect of oral administration of live LG2809 in a mouse model of oral tolerance induction. We found that LG2809 enhanced the suppression of proliferation and IL-2 production of SPL CD4^+^ T cells from OVA-fed DO11.10 mice. LG2809 increased the population of CD62L^low^ CD44^high^ CD4^+^ T cells, which was found to be anergic, to secrete a large amount of IL-10 and to have suppressive activity. Moreover, LG2809 increased the ratio of pDCs in the LP and enhanced their ability to induce regulatory T cells. These results suggested that LG2809 increased the population of pDCs in the LP and enhanced the induction of oral tolerance by increasing the ratio of CD62L^low^ CD44^high^ CD4^+^ T cells. To our knowledge, this is the first report to show how probiotics enhance the induction of oral tolerance.

The effect of probiotics on oral tolerance induction has been shown with *L*. *casei*. So *et al*. showed in a rat model of experimental arthritis that oral administration of *L*. *casei* suppresses arthritic inflammation by increasing the ratio of Foxp3^+^ CD4^+^ T cells, and increasing the secretion of IL-10 and TGF-β by CD4^+^ T cells [41]. We found an analogous increase in IL-10 production by the T cells but we did not observe an increase in the ratio of Foxp3^+^ T cells by oral administration of LG2809 in our experimental system. Moreover, LG2809 did not enhance TGF-β production (data not shown). Although it is difficult to compare the results because of differences in experimental conditions, we suggest that LG2809 might induce types of IL-10-producing Tr1 cells that are different from those induced by *L*. *casei*.

Oral administration of LG2809 increased the ratio of T cells with a CD62L^low^ CD44^high^ CD4^+^ effector phenotype. CD44 is a hyaluronan receptor and CD62L is L-selectin. Previous reports show that the expression of CD44 increases and the expression of CD62L decreases when CD4^+^ T cells are stimulated with antigen [54, 55]. Therefore, the loss of expression of CD62L is known as a marker of activation in CD4^+^ T cells. Taking these and our results into consideration, we suggest that feeding LG2809 enhances oral tolerance by promoting the proliferation and activation of antigen-specific CD4^+^ T cells.

In this study, we found that administering LG2809 led to an increase in the ratio of pDCs in the LP. Wittman *et al*. reported that feeding of *Bifidobacterium adolescentis* protected the infection of *Yersinia enterocolitica* probably by increase of pDC in intestine [56]. They discussed that the increase of pDCs by administration of *B*. *adolescentis* might depend on invariant NKT cells (iNKT). iNKT are responsible for the recruitment of pDCs to the pancreas during lymphocytic choriomeningitis virus infection [57]. Recently, it was reported that iNKT cells recognize glycolipid antigens from Gram-positive bacteria presented by CD1d [58]. The release of glycolipids by LG2809 might result in the activation of iNKT cells by which pDCs might be recruited to the intestine. Wang *et al*. reported that feeding mice the probiotic preparation VSL#3, which contains 8 strains of live bacteria namely *Bifidobacterium breve*, *Bifidobacterium longum*, *Bifidobacterium infantis*, *Lactobacillus acidophilus*, *Lactobacillus plantarum*, *Lactobacillus paracasei*, *Lactobacillus bulgaricus and Streptococcus thermophilus*, alters the population of DCs in the LP [27], but they observed a decrease in pDCs in the LP and found that the ratio of mDCs in the LP was greater in VSL#3-fed mice than in the controls. This result is the inverse of ours, suggesting that the effect of probiotics on the induction of LP DCs depends on the probiotic strains used. In addition, we found that regulatory CD4^+^ T cells differentiated through antigen presentation by LP cells from LG2809-administered mice produced a higher level of IL-10 and had stronger suppressive activity. Since LG2809 increased the ratio of pDCs but not mDCs in the LP, the enhancement of IL-10 production and T-cell suppressive activity of the CD4^+^ T cells was probably caused by the increased ratio of pDCs. The finding that pDCs induced IL-10-producing regulatory CD4^+^ T cells [26] strongly supports this hypothesis. It remains to be elucidated, however, whether an increase in LP pDCs can increase the population of anergic, IL-10 producing, and suppressive CD62L^low^ CD44^high^ CD4^+^ T cells in SPL.

Not only DC cells but also B cells can induce T cell tolerance via direct or indirect mechanisms [59, 60]. It is considered to be depending on experimental conditions whether B cells are essential for tolerance induction of T cells [59]. Oral tolerance of Th1 immune response was reported to be observed in B cell-depleted mice, suggesting that resting B cells are not essential for the induction of oral tolerance [61]. However, there are several studies showing B cells play a role in the oral tolerance induction [62]. In addition, chimeric mice specifically lacking IL-10 producing B cells were reported to develop an exacerbated collagen-induced arthritis and to have a reduction in IL-10 secreting Tr1 cells compared to wild type mice [63]. This suggests the importance of B cells in the differentiation and maintenance of Tr1 cells *in vivo*. It remains to be elucidated whether B cells are involved in the mechanisms by which LG2809 enhances the induction of oral tolerance.

Recently, the use of bispecific antibodies [64–66] and antibody multimers [67], and the use of regulatory T cells, Foxp3 Tregs [68] and Tr1 cells [69], have come to be proposed as a new modality to prevent and treat autoimmune and inflammatory diseases. Especially in the treatment of inflammatory bowel disease, Tr1 cells are believed to be a very promising because they have the strong anti-inflammatory effect [70]. Induction of antigen-specific Tr1 cells in vivo is possible using oral tolerance as described above. In fact, even up to now, application of the oral tolerance has been attempted to treat the autoimmune and inflammatory disease [12]. Probiotics, which promotes the induction of Tr1 cells in oral tolerance induction, may be useful in treating autoimmune and inflammatory diseases using oral tolerance.

In conclusion, we demonstrate that feeding LG2809 enhanced oral tolerance induction and suggest that the effect of LG2809 is exerted via an increase of pDCs in the LP and an increase of effector regulatory CD4^+^ T cells. The enhancement of oral tolerance induction by feeding LG2809 may be beneficial for the prevention of or early remission from food allergy.

## Supporting Information

S1 Fig The ratio of KJ-1.26^+^ CD62L^low^ Foxp3^-^ cells was correlated with IL-10 production in LG2809/OVA group. DO11.10 mice were treated as described in the legend of Fig 1. The correlation between the concentration of IL-10 in the culture supernatant of CD4^+^ T cells from LG2809/OVA group and the ratio of KJ-1.26^+^ CD62L^low^ Foxp3^-^ (A) and KJ-1.26^+^ CD62L^low^ Foxp3^+^ cells (B) among CD4^+^ T cells from LG2809/OVA group. R, Pearson correlation coefficient.
(TIFF)
