# Supplementary figures and images for: Enhancement of Oral Tolerance Induction in DO11.10 Mice by Lactobacillus gasseri OLL2809 via Increase of Effector Regulatory T Cells
Source: PLoS One. 2016 Jul 29;11(7):e0158643. doi: 10.1371/journal.pone.0158643 (PMC4966961; doi:10.1371/journal.pone.0158643)

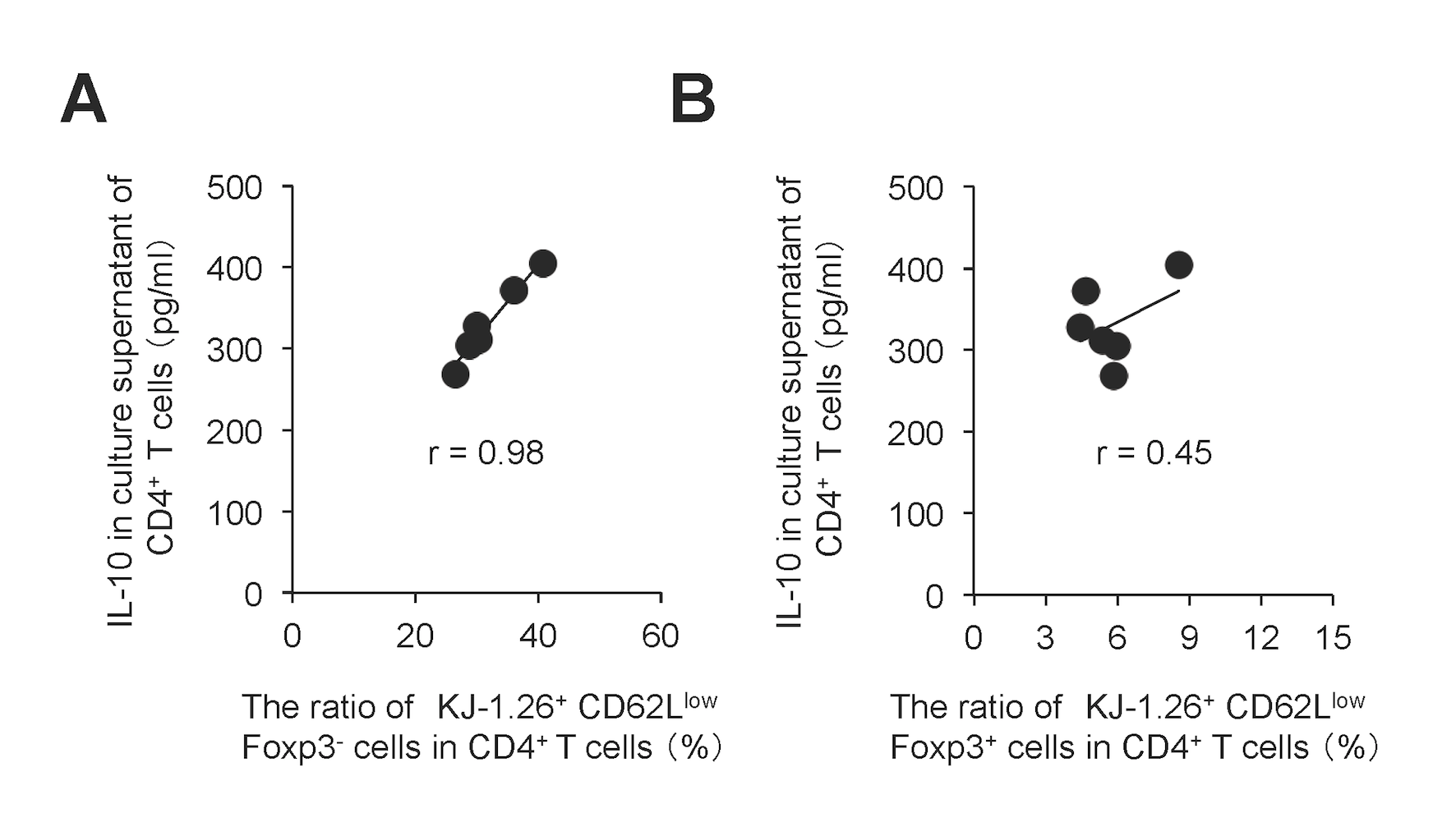

Supplement: S1 Fig — DO11.10 mice were treated as described in the legend of Fig 1. The correlation between the concentration of IL-10 in the culture supernatant of CD4+ T cells from LG2809/OVA group and the ratio of KJ-1.26+ CD62Llow Foxp3- (A) and KJ-1.26+ CD62Llow Foxp3+ cells (B) among CD4+ T cells from LG2809/OVA group. R, Pearson correlation coefficient. (TIFF) [file pone.0158643.s001.tiff]
